# Under a nonadherent state, bone marrow mesenchymal stem cells can be efficiently induced into functional islet-like cell clusters to normalize hyperglycemia in mice: a control study

**DOI:** 10.1186/scrt455

**Published:** 2014-05-08

**Authors:** Yihua Zhang, Zhongying Dou

**Affiliations:** 1 Shaanxi Branch of National Stem Cell Engineering and Technology Centre, College of Veterinary Medicine, Northwest A & F University, Yangling, Shaanxi 712100, China

## Abstract

**Introduction:**

Bone marrow mesenchymal stem cells (BMSCs) possess low immunogenicity and immunosuppression as an allograft, can differentiate into insulin-producing cells (IPCs) by *in vitro* induction, and may be a valuable cell source to regenerate pancreatic islets. However, the very low differentiation efficiency of BMSCs towards IPCs under adherent induction has thus far hindered the clinical exploitation of these cells. The aim of this study is to explore a new way to efficiently induce BMSCs into IPCs and lay the groundwork for their clinical exploitation.

**Methods:**

In comparison with adherent induction, BMSCs of human first-trimester abortus (hfBMSCs) under a nonadherent state were induced towards IPCs in noncoated plastic dishes using a three-stage induction procedure developed by the authors. Induction effects were evaluated by statistics of the cell clustering rate of induced cells, and ultrastructural observation, dithizone staining, quantitative polymerase chain reaction and immunofluorescence assay, insulin and c-peptide release under glucose stimulus of cell clusters, as well as transplantation test of the cell clusters in diabetic model mice.

**Results:**

With (6.175 ± 0.263) × 10^5^ cells in 508.5 ± 24.5 cell clusters, (3.303 ± 0.331) × 10^5^ single cells and (9.478 ± 0.208) × 10^5^ total cell count on average, 65.08 ± 2.98% hfBMSCs differentiated into pancreatic islet-like cell clusters after nonadherent induction. With (3.993 ± 0.344) × 10^5^ cells in 332.3 ± 41.6 cell clusters, (5.437 ± 0.434) × 10^5^ single cells and (9.430 ± 0.340) × 10^5^ total cell count on average, 42.37 ± 3.70% hfBMSCs differentiated into pancreatic islet-like cell clusters after adherent induction (*P* < 0.01, *n* = 10). The former is significantly higher than the latter. Calculated according to the cell clustering rate and IPC percentage in the cell clusters, 29.80 ± 3.95% hfBMSCs differentiated into IPCs after nonadherent induction and 18.40 ± 2.08% hfBMSCs differentiated into IPCs after adherent induction (*P* < 0.01, *n* = 10), the former significantly higher than the latter. The cell clusters expressed a broad gene profile related to pancreatic islet cells, released insulin and c-peptide in a glucose concentration-dependent manner, and normalized hyperglycemia of streptozocin-induced mice for at least 80 days following xenograft. Blood glucose of grafted mice rose again after their graft removed. A series of examination of the grafts showed that transplanted cells produced human insulin in recipients.

**Conclusions:**

Our studies demonstrate that nonadherent induction can greatly promote BMSCs to form pancreatic islet-like cell clusters, thereby improving the differentiation efficiency of BMSCs towards IPCs.

## Introduction

Type 1 diabetes (insulin-dependent diabetes mellitus), with an annual net rise of about 3% in incidence [1], results from the autoimmune lesion of pancreatic beta-cells. Insulin-dependent diabetes mellitus has been successfully treated by transplantation with pancreatic islets of Langerhans [2,3], but further application of this therapy is limited by the lack of Langerhans donors and immunological rejection. Fortunately, bone marrow mesenchymal stem cells (BMSCs) not only differentiate into multiple mesodermal and nonmesodermal cell lineages including insulin-producing cells (IPCs) [4-11], but also possess low immunogenicity [12] and immunosuppression by modulating the immune function of the major cell populations involved in alloantigen recognition and elimination [13-21]. These unique biological properties make BMSCs a promising source for cell replacement of kinds of tissue necrosis and function cell exhaustion, especially insulin-dependent diabetes mellitus.

Under adherent induction, however, BMSCs are not effective to form cell clusters, leading to the very low differentiation efficiency of BMSCs towards IPCs. We hypothesized that nonadherent induction favors the formation of cell clusters, thereby improving the differentiation efficiency of BMSCs towards IPCs. To verify this hypothesis, we induced BMSCs of human first-trimester abortus (hfBMSCs) under a nonadherent state to efficiently differentiate into functional islet-like cell clusters, which corrected hyperglycemia in model mice following xenograft. This study provides a new way to induce BMSCs *in vitro* into pancreatic islet-like cells to treat insulin-dependent diabetes mellitus.

## Methods

### Preparation of hfBMSCs

Under permission of the patients, a local hospital and the Ethics Committee of Northwest A & F University, hfBMSCs were isolated from long bones of 2-month-old to 3-month-old human abortuses using whole-marrow cell culture and proliferated in α-modified Eagle’s medium (Gibco, Billings, Montana, USA), 10% fetal calf serum (Stemcell Technologies Inc., Vancouver, British Columbia, Canada) and 0.1 mmol/l β-mercaptoethanol (Sigma Loveland, CO, USA). The cells were identified using flow cytometry (Beckman Coulter Inc., Fullerton, California, USA) and CD29, CD44, CD166, CD11a, CD14 and CD34 fluorescence-tagged antibodies (Beckman Coulter Inc.).

### *In vitro* induction of hfBMSCs towards insulin-producing cells

Passage 6 of the cryopreserved hfBMSCs was thawed, and proliferated to passage 8 in α-modified Eagle’s medium, 20% fetal calf serum, and 0.1 mmol/l β-mercaptoethanol. Passage 8 of hfBMSCs underwent acclimation in Dulbecco’s modified Eagle’s medium (DMEM)–high glucose (containing 25 mmol/l glucose; HyClone, Logan, Utah, USA), 10% fetal calf serum, and 0.1 mmol/l β-mercaptoethanol, were digested, were transferred into noncoated plastic dishes (in which hfBMSCs are nonadherent), and were induced using a three-stage induction procedure developed by the authors. This procedure was respectively performed 10 times using hfBMSCs from different abortus (*n* = 10), with nine replications each and each replication covering one dish containing more than 1 × 10^6^ hfBMSCs, as the nonadherent induction group. The same hfBMSCs were transferred into lysine-coated plastic dishes (in which hfBMSCs are adherent) and induced using the same procedure (*n* = 10) as the adherent induction group. 

In stage one of the procedure, the hfBMSCs were cultured for 6 days to develop nestin-positive cells with DMEM–high glucose plus 10 ng/ml basic fibroblast growth factor (Invitrogen, Carlsbad, California, USA), 10 ng/ml epidermal growth factor (EGF; Chemicon, California, USA), 2% B27 supplement (Stemcell Technologies Inc.), 0.5% bovine serum albumin (BSA; Invitrogen), and 0.1 mmol/l β-mercaptoethanol, with medium changes every 2 days. Stage two differentiated nestin-positive cells into IPCs via a 6-day induction with DMEM–high glucose plus 10 ng/ml EGF, 20 ng/ml Activin A (Sigma), 10 mmol/l nicotinamide (Sigma), 2% B27, 0.5% BSA, and 0.1 mmol/l β-mercaptoethanol, with medium changes every 2 days. Stage three was the maturation of the expected IPCs via a 4-day culture with DMEM–low glucose (containing 5.6 mmol/l glucose; Gibco) plus 10 ng/ml EGF, 10 nmol/l exendin-4 (Sigma), 10 ng/ml betacellulin (Sigma), 2% B27, 0.5% BSA, and 0.1 mmol/l β-mercaptoethanol, with medium changes every 2 days. 

### Statistics of the cell clustering rate

After each time induction, one dish was picked out randomly, cell clusters and single cells in the dishes were collected respectively, and cells from the cell clusters and the single cells were counted respectively. The cell clustering rate was calculated according to the cell number in the cell clusters divided by the summation of the single cells and cells in the cell clusters (*n* = 10), and the average cell number of each cell cluster was calculated according to the cell number in the cell clusters divided by the cell cluster number (*n* = 10). 

### Ultrastructural observation of endocrine cells in cell clusters

After each time induction, one dish was randomly picked from the nonadherent induction group and the adherent induction group (*n* = 10). The cell clusters were centrifuged for 10 minutes, fixed in 0.1% glutaraldehyde/2% formaldehyde in 0.1 mol/l cacodylate buffer, transferred to 0.1 mol/l cacodylate buffer, and embedded. Ultrathin sections were prepared, observed and photographed under a transmission electron microscope (H-7650; Hitachi, Japan). Fetal pancreatic islets, newly isolated from the pancreas of human abortus at the age of 20 weeks according to Kover and Moore [22], were used as the positive control.

### Dithizone staining

After each time induction, one dish was randomly picked from the nonadherent induction group and the adherent induction group and cell clusters were collected respectively (*n* = 10). The cell clusters, non-induced hfBMSCs (as non-induction control), and some fetal pancreatic islets (as positive control) were stained for 15 minutes with a freshly prepared dithizone (Sigma) working solution at 37°C, washed three times with phosphate-buffered saline (PBS), observed and photographed under an optical microscope.

### Fluorescent quantitative reverse transcriptase-polymerase chain reaction assay

To clarify the *in vitro* expression levels of pdx1, ngn3, pax4, neuroD1, nkx2.2, nkx6.1, PCSK1, insulin, glucagon, SST, and PP genes in induced cells, fluorescent quantitative reverse transcriptase-polymerase chain reaction was performed. Total RNA of cell clusters from each time induction in the nonadherent induction group and the adherent induction group, fetal pancreatic islets as positive control and non-induced hfBMSCs as non-induction control were extracted with TRlzol® Reagent (Invitrogen) and each was reverse-transcribed into cDNA with the PrimerScript RT reagent kit (TaKaRa, Tokyo, Japan) according to the manufacturer’s manual (*n* = 10). The forward and reverse primers and the fluorescent quantitative polymerase chain reaction details for the marker genes in this study are presented in Table 1.

**Table 1:** Human gene-specific primers and quantitative polymerase chain reaction conditions

**Gene**	**Primer sequence (5′ to 3′)**	**Size of PCR product (base pairs)**	**Annealing temperature (°C)**	**Cycle number**
18SrRNA	Forward: CGGCTACCACATCCAAGGAA	187	62	40
	Reverse: GCTGGAATTACCGCGGCT			
Pdx1	Forward: TTCCGGAAGAAAAAGAGCCA	129	62	40
	Reverse: AAACAGGTCCCAAGGTGGAGT			
Ngn3	Forward: CGCAATCGAATGCACAACCT	135	62	40
	Reverse: TTGAGTCAGCGCCCAGATGTA			
Pax4	Forward: ATTCAGTGGCCCGTGGAAA	102	62	40
	Reverse: TCTCTTGCCGACGCCATTT			
NeuroD1	Forward: TCTTTCAAACACGAACCGTCC	103	62	40
	Reverse: AGATTGATCCGTGGCTTTGG			
Nkx2.2	Forward: GCCACGAATTGACCAAGTGAA	103	62	40
	Reverse: ATGTCCTTGACCGAAAACCCC			
Nkx6.1	Forward: ACACGAGACCCACTTTTTCCG	102	62	40
	Reverse: AATAGGCCAAACGAGCCCTCT			
PCSK1	Forward: TCCTCTTTTGCGCTTGGTGT	139	62	40
	Reverse: TGACCCAAAAGGTCATAGCCC			
Insulin	Forward: GCGTGGCATTGTGGAACAA	123	62	40
	Reverse: CCATCTCTCTCGGTGCAGGA			
Glucagon	Forward: ACATTGCCAAACGTCACGATG	103	62	40
	Reverse: GCAATGAATTCCTTGGCAGCT			
SST	Forward: GAAGCAGGAACTGGCCAAGTA	118	62	40
	Reverse: CCTCATTTCATCCTGCTCAGC			
PP	Forward: ACAATGCCACACCAGAGCAGA	110	62	40
	Reverse: GGCCAGCGTGTCCTCTTTG			

PCR, polymerase chain reaction.

### Immunofluorescence assay

To test the expression of nestin, insulin and c-peptide during the induction, cell clusters were sampled individually following each induction stage, respectively fixed in 4% paraformaldehyde for 20 to 30 minutes, triple-washed with PBS, and stained with mouse antibodies against human nestin (Abcam, Cambridge, Massachusetts, UK) and insulin (Invitrogen), and rabbit anti-human c-peptide antibody (Abcam), and then with secondary fluorescent antibodies including fluorescein isothiocyanate-conjugated donkey anti-mouse IgG and Alexa Flour 594-conjugated goat anti-rabbit IgG (Invitrogen). To investigate the expression rate of IPCs and other endocrine cells in cell clusters, the cell clusters from each time induction were respectively digested, subcultured for 24 hours in a lysine-coated 24-well plate before fixation, stained with mouse anti-human insulin and glucagon, rabbit anti-human SST and PP antibodies (Invitrogen), and the relevant IgGs conjugated with fluorescence. Ten nonoverlapping visual fields were randomly chosen from each staining sample to count stained and nonstained cells under microscopy, and they were photographed using an inverted microscope. The proportion of cells immunoreactive to particular antigens was quantified according to the stained cell number divided by the summation number of stained cells and nonstained cells (*n* = 10).

### *In vitro* insulin and c-peptide release in response to increasing glucose concentrations

After each time induction, cell clusters were sampled from the nonadherent induction group and the adherent induction group respectively, transferred into 12-well culture plates containing a lysine coating for cells to attach, 100 clusters per well, and were precultured in DMEM–low glucose, 10 ng/ml EGF, 2% B27, 0.5% BSA, and 0.1 mmol/l β-mercaptoethanol for 24 hours, washed three times with PBS, and stimulated with 1 ml of either 5, 10, or 25 mmol/l glucose in PBS containing 1% BSA (*n* = 10). In parallel, 100 fetal pancreatic islets (positive control) and 1.2 × 10^5^ hfBMSCs at passage 8 (equivalent to 100 cell clusters, non-induction control) were treated in the same way with all three glucose levels. The supernatants from all treatments were collected after 30 minutes of stimulation, and insulin and c-peptide were measured via radioimmunoassay with the human insulin-specific RIA kit and the human c-peptide RIA kit (LINCO Research Inc., St. Charles, MO, USA) and a liquid scintillation counter (LS6000; Beckman Co., USA).

### Preparation of type 1 diabetic model mice

Approved by the Institutional Animal Care and Use Committee of Northwest A & F University, a total of 35 adult male nude mice (BALB/c nu/nu), provided by the Experiment Animal Centre of the Fourth Military Medical University (Xi’an, China), each with normal blood glucose levels, were administered daily intraperitoneal injections of 50 mg/kg streptozocin (STZ; Sigma) for 5 consecutive days. At 72 hours after the last STZ injection, fasting blood glucose levels were determined using a standard blood glucose meter (Sure Step™ Plus; LifeScan Inc., Milpitas, CA, USA) once a day for 3 consecutive days. If the three fasting blood glucose levels were all higher than 18 mmol/l, the mouse was used as a diabetic model animal for our experiments.

### Transplantation of CM-DiI-labeled cell clusters to treat diabetic mice

Cell clusters from the nonadherent induction group and the adherent induction group, and also non-induced hfBMSCs, were incubated individually in a working solution of Cell Tracker–CM-DiI (Molecular Probes, Invitrogen Carlsbad, California, USA) for 5 minutes at 37°C and for another 15 minutes at 4°C according to the manufacturer’s instructions, followed by washing with PBS, and were checked under a fluorescence microscope. The 600 CM-DiI-labeled cell clusters from the nonadherent induction (about 7.2 × 10^5^ cells) were transplanted into the right-side testis of 10 diabetic model mice each as the nonadherent induction group (*n* = 10). The 600 CM-DiI-labeled cell clusters from the adherent induction were transplanted into the right-side testis of another 10 diabetic model mice each as the adherent induction group (*n* = 10). CM-DiI-labeled but non-induced 7.2 × 10^5^ hfBMSCs were transplanted into the right-side testis of another six diabetic model mice each as the non-induction control group (*n* = 6). Blood glucose levels of animals in each of the three treatments were measured once every 4 days using a standard blood glucose meter and the body weight was measured once every 8 days using an electronic balance (Mettler-Toledo Inc., Columbus, OH, USA).

To examine the effects of graft removal on the blood glucose fluctuation and the survival situation of the recipients, and the *in situ* insulin production of the xenografts, three mice were randomly selected from each animal group and their right testes removed 28 days post transplantation following similar blood glucose and body weight measurements. Histological sections of all testicular grafts were dewaxed and stained with mouse monoclonal antibodies against human insulin and then with fluorescein isothiocyanate-conjugated donkey anti-mouse IgG, and were examined using a fluoroscope.

To evaluate the glucose clearance effects of the transplanted islet-like cell clusters, the intraperitoneal glucose tolerance test was performed on five mice in each of the nonadherent induction group and the adherent induction group after 12 days of glucose level normalization following the transplantation and on five nondiabetic mice (as normal control). Each mouse was intraperitoneally injected with glucose at 2 mg/g body weight under fasting conditions and tested for blood glucose at 0, 30, 60, 90, 120, and 150 minutes thereafter [23].

### Data processing

Data are treated for significance with Student’s *t* test or SPSS 12.0 simplified Chinese version one-way analysis of variance where applicable. In all cases, all values are presented as mean ± standard deviation and *P* < 0.05 was considered significant.

## Results

### Preparation of hfBMSCs

**Figure 1:**
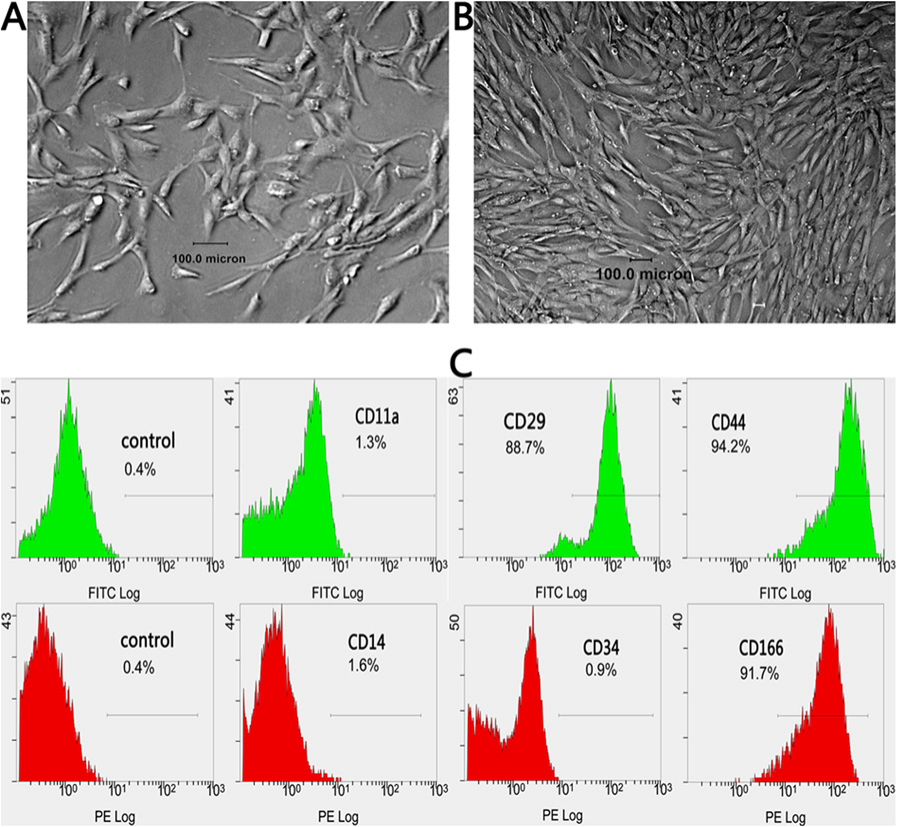
**Isolation and identification of bone marrow mesenchymal stem cells of human first-trimester abortus.** Adhered spindle cells appeared in culture of whole bone marrow **(A)** and they clonally grew and spread at the bottom of a culture dish within the 12th day **(B)**. Flow cytometric analysis showed that passage 6 of the isolated cells strongly expressed the surface markers of mesenchymal stem cells, such as CD29, CD44 and CD166, but almost no markers of Hematopoietic Stem Cells, such as CD11a, CD14 and CD34 **(C)**. FITC, fluorescein isothiocyanate; PE, phycoerythrin.

The whole-marrow cell culture resulted in spindle cell adherence (Figure 1A). Many spindle cells then clonally grew and spread at the bottom of a culture dish within the 12th day (Figure 1B). Several hundred dishes of sixth-passage cells (about 10^6^ cells in every dish) were obtained upon continuously passaging culture and were cryopreserved in liquid nitrogen. Flow cytometric analysis showed that passage 6 of the isolated cells strongly expressed the surface markers of mesenchymal stem cells, such as CD29, CD44 and CD166, but almost no markers of Hematopoietic Stem Cells, such as CD11a, CD14 and CD34 (Figure 1C).

### Differentiation of hfBMSCs into islet-like cell clusters

**Figure 2:**
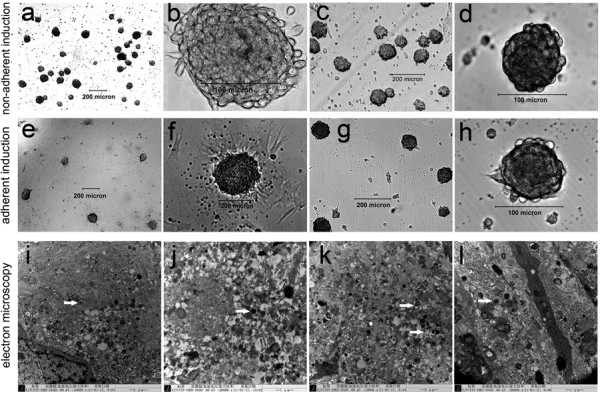
**Differentiation of bone marrow mesenchymal stem cells of human first-trimester abortus into islet-like cell clusters.** Many irregular cell clusters formed in the first-stage induction **(A)**, became compact in the second-stage induction **(B)**, and became spherical with blurred boundaries among adjacent cells in the third-stage induction, similar to the morphology of the islets of Langerhans, whereas some of the nonclustered cells adhered to the dish bottom and became similar to bone marrow mesenchymal stem cells of human first-trimester abortus in shape in the nonadherent induction group **(C)**, **(D)**. Irregular cell clusters after the first-stage induction **(E)** became hemispherical in the second-stage induction **(F)**, uplifted with blurred boundaries among adjacent cells in the third-stage induction in the adherent induction group **(G)**, **(H)**, and always adhered to the dish bottom. Transmission electron microscopy showing a large number of endocrine cells **(I)** and secretion granules in cytoplasma of the cells in the cell clusters from the nonadherent induction group **(J)** and the adherent induction group **(K)** after the three-stage induction, histologically similar to those in fetal pancreatic islets as the positive control **(L)**.

In the first-stage induction, most hfBMSCs in the nonadherent induction group congregated on the dish bottoms to gradually form many irregular cell clusters (Figure 2A), whereas a few cells died and floated on the medium surface. Comparatively, less than one-half of the hfBMSCs in the adherent induction group congregated into irregular cell clusters adhering to the dish bottom (Figure 2E). In the second-stage induction, the cell clusters in the nonadherent induction group gradually became compact (Figure 2B), whereas the cell clusters in the adherent induction group became hemispherical and firmly adhered to the dish bottom (Figure 2F). In the third-stage induction, the cell clusters in the nonadherent induction group gradually became spherical with blurred boundaries between adjacent cells on the surface, similar to the morphology of the islets of Langerhans, whereas some of the nonclustered cells adhered to the dish bottom and became similar to hfBMSCs in shape (Figure 2C,D). Comparatively, the cell clusters in the adherent induction group gradually uplifted but still adhered to the dish bottom, with blurred boundaries between adjacent cells on the surface (Figure 2G,H).

With (6.175 ± 0.263) × 10^5^ cells in 508.5 ± 24.5 cell clusters, (3.303 ± 0.331) × 10^5^ single cells, (9.478 ± 0.208) × 10^5^ total cell count and 1,215.07 ± 35.38 cells in each cluster on average, 65.08 ± 2.98% hfBMSCs differentiated into pancreatic islet-like cell clusters in the nonadherent induction group (*n* = 10). With (3.993 ± 0.344) × 10^5^ cells in 332.3 ± 41.6 cell clusters, (5.437 ± 0.434) × 10^5^ single cells, (9.430 ± 0.340) × 10^5^ total cell count and 1,199.36 ± 63.41 cells in each cluster on average, 42.37 ± 3.70% hfBMSCs differentiated into pancreatic islet-like cell clusters in the adherent induction group (*n* = 10). The former is significantly higher than the latter (*P* < 0.01, *n* = 10). However, the total cell count and average cell number of each cell cluster are not significantly different between the nonadherent induction group and the adherent induction group (*P* > 0.05, *n* = 10). Under transmission electron microscopy, a large number of endocrine cells (Figure 2I) and secretion granules in cytoplasma were observed in the sections of islet-like cell clusters in the nonadherent induction group (Figure 2J) and the adherent induction group (Figure 2K), which were histologically similar to those in fetal pancreatic islets as a positive control (Figure 2L).

### Dithizone staining

**Figure 3:**
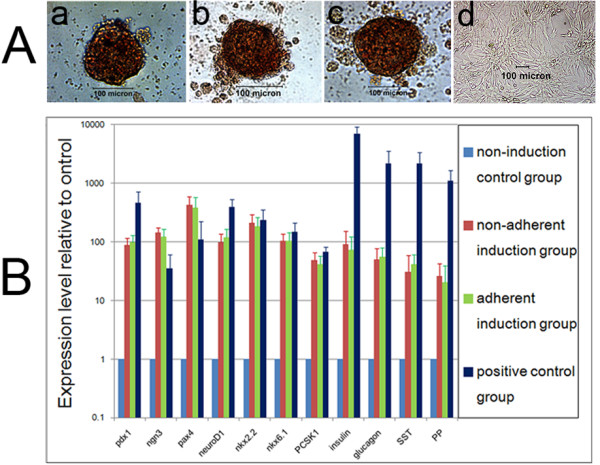
**Dithizone staining and quantitative polymerase chain reaction analysis of islet-like cell clusters from bone marrow mesenchymal stem cells of human first-trimester abortus. (A)** Islet-like cell clusters were positive for dithizone staining in the nonadherent induction group **(a)** and the adherent induction group **(b)**, similar to those of fetal pancreatic islets **(c)**, whereas non-induced bone marrow mesenchymal stem cells of human first-trimester abortus were negative for dithizone **(d)**. **(B)** Expression levels of Pdx1, Ngn3, Pax4, NeuroD1, Nkx2.2, Nkx6.1, PCSK1, insulin, glucagon, SST and PP genes in fluorescent quantitative reverse transcriptase-polymerase chain reaction test. The islet-like cell clusters expressed significantly more Ngn3 and Pax4, and significantly less insulin, glucagon, SST and PP than fetal pancreatic islets (*P* < 0.01, *n* = 10). The expressions of all the genes were not different between the nonadherent induction group and the adherent induction group (*P* > 0.05, *n* = 10).

Dithizone is a zinc-chelating agent used to selectively stain pancreatic beta-cells crimson red. The insulin production of the islet-like cell clusters was verified by positive dithizone staining in the nonadherent induction group (Figure 3A.a) and the adherent induction group (Figure 3A.b), similar to staining behavior of fetal pancreatic islets (Figure 3A.c), and non-induced hfBMSCs were negative for dithizone (Figure 3A.d).

### Fluorescent quantitative reverse transcriptase-polymerase chain reaction

Fluorescent quantitative reverse transcriptase-polymerase chain reaction (Figure 3B) showed that the islet-like cell clusters in the nonadherent induction group and the adherent induction group all expressed pdx1, ngn3, pax4, neuroD1, nkx2.2, nkx6.1, PCSK1, insulin, glucagon, SST and PP, similar to the expression profile of fetal pancreatic islets. However, the islet-like cell clusters in the nonadherent induction group and the adherent induction group expressed significantly more Ngn3 and Pax4, and significantly less insulin, glucagon, SST and PP than fetal pancreatic islets (all *P* < 0.01, *n* = 10), indicating that the cell clusters from hfBMSCs induced *in vitro* were not mature enough. The expression of the genes was similar between the nonadherent induction group and the adherent induction group.

### Immunofluorescence assay

**Figure 4:**
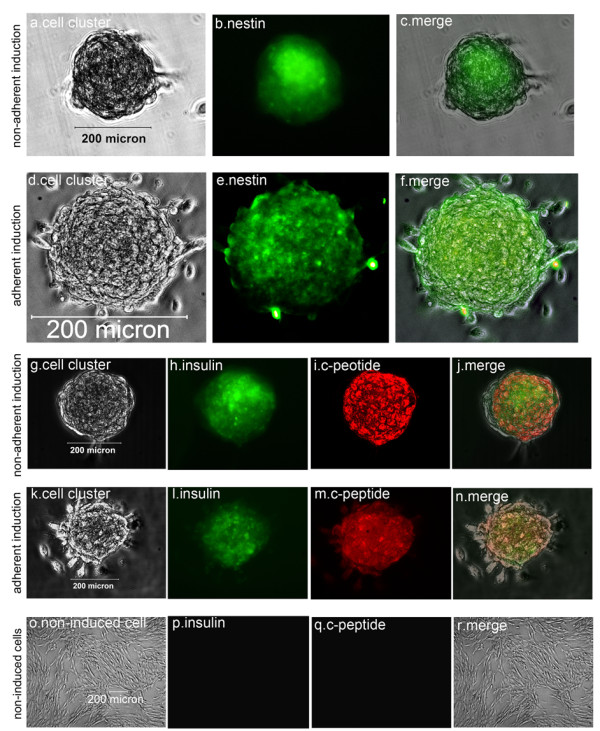
**Immunofluorescence assay of whole islet-like cell clusters.** The strongest expression of nestin is in islet-like cell clusters after the first-stage induction in the nonadherent induction group **(A), (B), (C)** and the adherent induction group **(D), (E), (F)**. The strongest expression of insulin and c-peptide is in islet-like cell clusters after the third-stage induction in the nonadherent induction group **(G), (H), (I), (J)** and the adherent induction group **(K), (L), (M), (N)**. Non-induced bone marrow mesenchymal stem cells of human first-trimester abortus were negative for insulin and c-peptide **(O), (P), (Q), (R)**.

**Figure 5:**
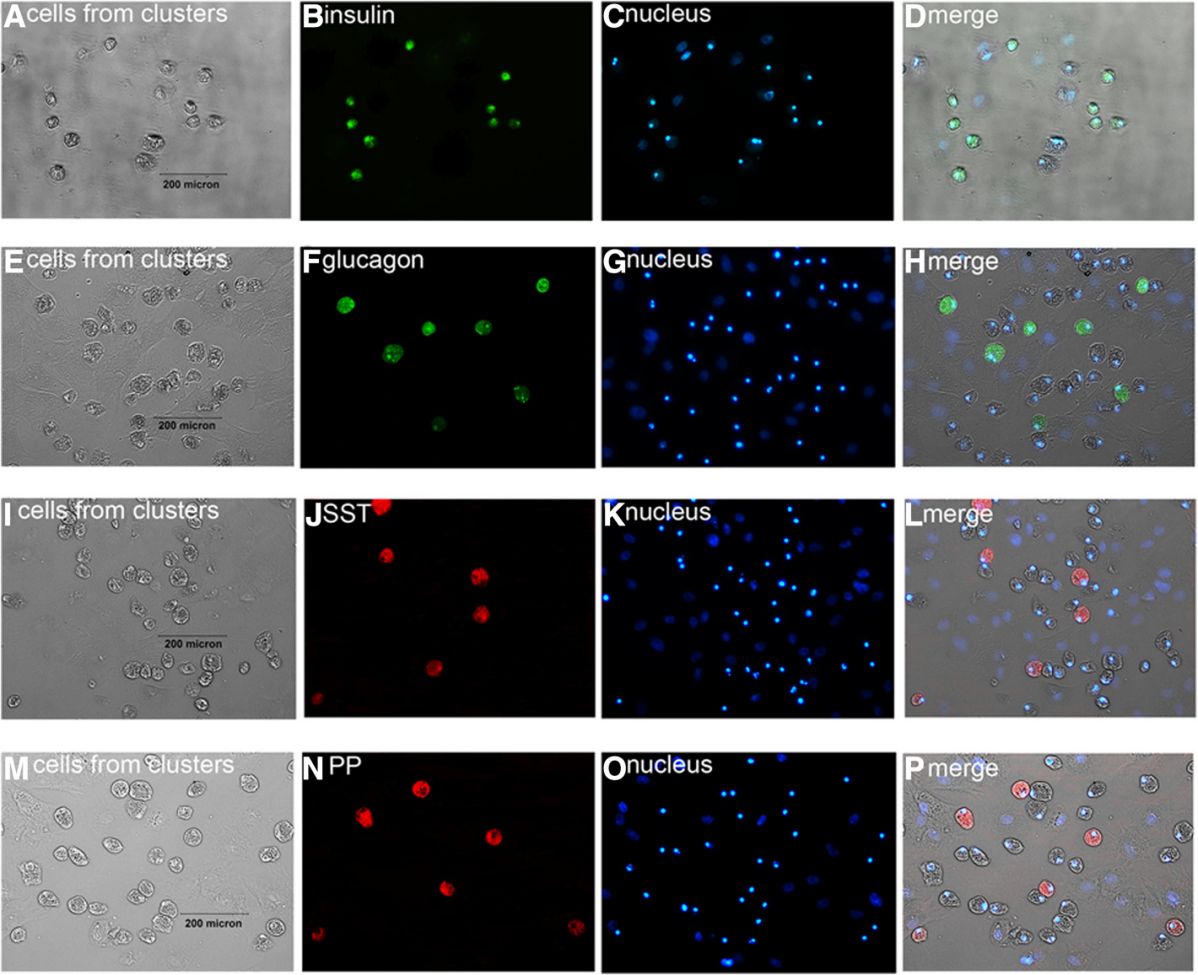
**Immunofluorescence assay of single cells from islet-like cell clusters.** On enzymatic dispersion and reculture in lysine-coated plastic dishes, the single cells from islet-like cell clusters adhered and expressed insulin **(A), (B), (C), (D)**, glucagon **(E), (F), (G), (H)**, SST **(I), (J), (K), (L)** and PP **(M), (N), (O), (P)** respectively.

The immunofluorescence assay indicated that the islet-like cell clusters from the nonadherent induction group and the adherent induction group all expressed nestin, insulin and c-peptide (Figure 4); that is, nestin was more expressed after the first-stage induction in the nonadherent induction group (Figure 4A,B,C) and the adherent induction group (Figure 4D,E,F), and insulin and c-peptide more expressed after the third-stage induction in the nonadherent induction group (Figure 4G,H,I,J) and the adherent induction group (Figure 4K,L,M,N) but did not express after the first-stage induction. In contrast, non-induced hfBMSCs did not express insulin and c-peptide (Figure 4O,P,Q,R). The single cells from the digestion of islet-like cell clusters following the three-stage induction adhered during 24 hours of subculture and then were fixed and stained for insulin, glucagon, SST and PP (Figure 5). The positive cell percentages for insulin, glucagon, SST and PP were successively 45.79 ± 5.87%, 7.12 ± 2.15%, 6.33 ± 2.86% and 4.54 ± 1.54% in the nonadherent induction group (*n* = 10), and were 43.42 ± 6.81%, 8.35 ± 2.72%, 5.38 ± 2.13% and 3.66 ± 1.32% in the adherent induction group (*n* = 10) respectively. The percentages of each kind of positive cells were not different between the nonadherent induction group and the adherent induction group (all *P* > 0.05, *n* = 10). In addition, the differentiation rate of hfBMSCs towards IPCs (the cell clustering rate **×** the percentage of IPCs in islet-like cell clusters) was calculated as 29.80 ± 3.95% in the nonadherent induction group, which was significantly higher than the differentiation rate (18.40 ± 2.08%) in the adherent induction group (*P* < 0.01, *n* = 10).

### *In vitro* insulin and C-peptide release in response to a glucose challenge

**Figure 6:**
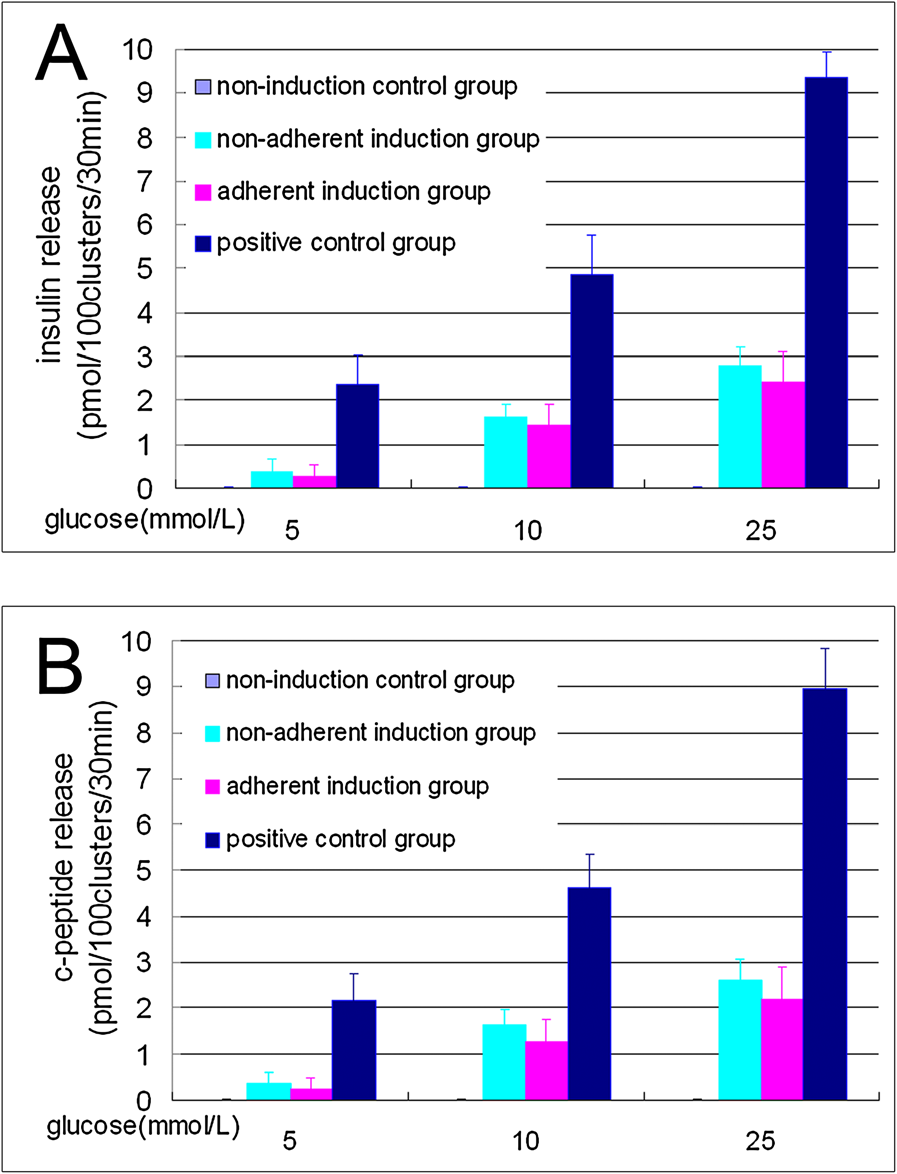
**Insulin and C-peptide release by islet-like cell clusters.** Radioimmunoassay results show that the release of insulin **(A)** and c-peptide **(B)** is significantly different between the nonadherent induction group and the non-induction control group, between the nonadherent induction group and the positive control group, and between the different glucose levels in the nonadherent induction group (all at *P* < 0.01, *n* = 10), but were not different between the nonadherent induction group and the adherent induction group (*P* > 0.05, *n* = 10).

The results of glucose challenge (Figure 6) indicated that the islet-like cell clusters from the nonadherent induction group and the adherent induction group all released insulin and c-peptide at significantly higher levels than those of the non-induction control group (all *P* < 0.01, *n* = 10). The results also indicated that the insulin and c-peptide releases of the islet-like cell clusters in response to glucose challenge occurred in a concentration-dependent manner because there was significant difference of each release among different glucose levels (all *P* < 0.01, *n* = 10). Finally, the release of insulin and c-peptide from the islet-like cell clusters in the nonadherent induction group and the adherent induction group was much less than those of fetal pancreatic islets (all *P* < 0.01, *n* = 10), and they were no different between the nonadherent induction group and the adherent induction group (all *P* > 0.05, *n* = 10).

### Effects of islet-like cell clusters to treat diabetic mice

**Figure 7:**
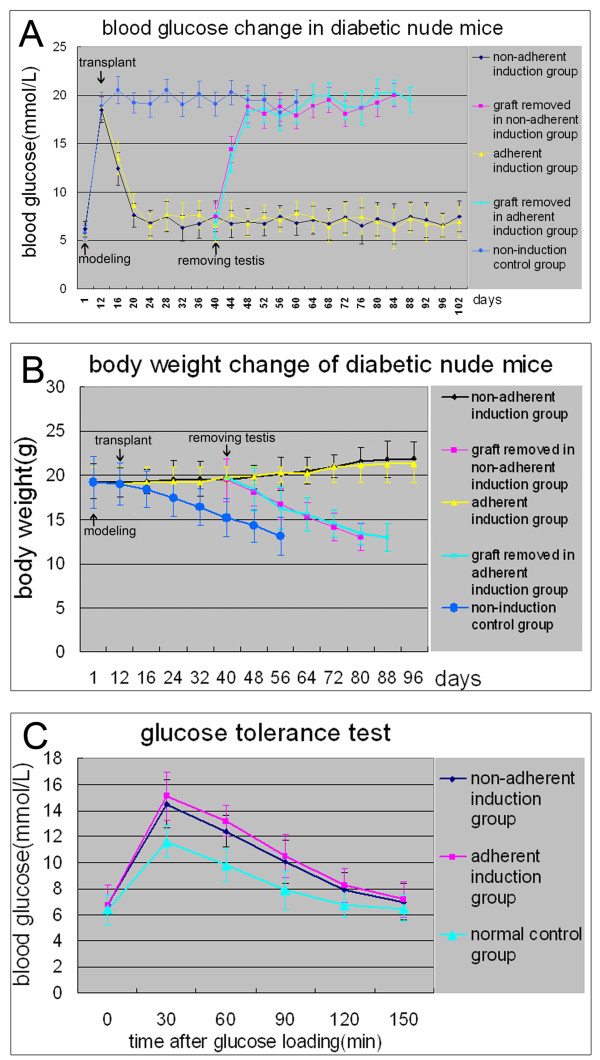
**Effects of xenograft with islet-like cell clusters on the recovery of streptozocin-induced diabetic mice.** In the nonadherent induction group, the blood glucose levels of 10 diabetic mice fell to normal within 2 weeks following the transplantation, but three of the 10 mice regained hyperglycemia when their xenograft was removed 28 days post transplantation and all died within 45 days after the removal; the remaining seven mice without the removal of xenograft maintained normal levels of blood glucose for at least 80 days **(A)** and gained body weight slightly **(B)**. The intraperitoneal glucose tolerance test indicated that the islet-like cell clusters had a normal glucose clearance rate after transplantation, but were not as effective as native pancreatic beta-cells **(C)**. The effects of xenograft in the adherent induction group are similar to those in the nonadherent induction group. In the non-induction control group, six diabetic mice maintained high blood glucose levels (>18 mmol/l) after bone marrow mesenchymal stem cell of human first-trimester abortus transplantation, lost their body weight continuously and died within 45 days after the transplantation, and so did the mice with removal of their testes 28 days post transplantation.

After STZ injection, 26 of 35 (74%) of the nude mice became eligible diabetic models. Following transplantation with islet-like cell clusters, in the nonadherent induction group the blood glucose levels of 10 diabetic mice all sank to normal levels within 12 days, but the blood glucose levels of three mice whose testes was removed 28 days after transplantation rose again and they all died within another 45 days; the remaining seven mice without the removal of xenograft maintained normal blood glucose values for at least 80 days (Figure 7A) and gained body weight slightly (Figure 7B). The transplantation effects in the adherent induction group were similar to those in the nonadherent induction group. In the non-induction control group, six diabetic mice maintained high blood glucose levels (>18 mmol/l) after hfBMSC transplantation, lost their body weight continuously and died within 45 days after the transplantation, and so did the three mice with removal of their testes 28 days post transplantation.

**Figure 8:**
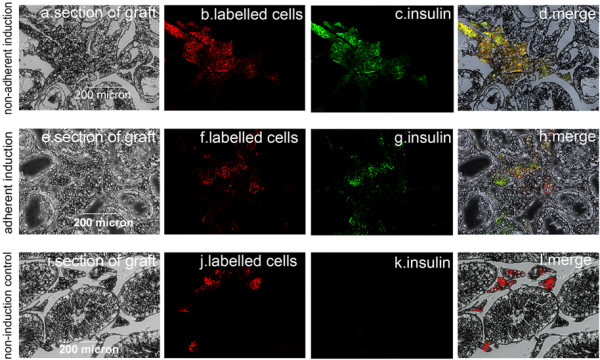
**Immunofluorescence examination of dewaxed sections of the grafts.** Red CM-DiI-labeled cells in a graft section **(A)** in the nonadherent induction group were visualized only in the interstitial tissue among the seminiferous tubules **(B)** and showed green fluorophore **(C)** (merged in **(D)**) after staining with human insulin antibody under a fluoroscope. A similar phenomenon occurred in a graft section in the adherent induction group **(E), (F), (G), (H)**, whereas CM-DiI-labeled cells in the non-induction control group did not express insulin **(I), (J), (K), (L)**.

The results of the intraperitoneal glucose tolerance test (Figure 7C) indicated that the islet-like cell clusters from the nonadherent induction group and the adherent induction group were functional, and they were indeed responsive to a glucose challenge *in vivo*, but were not as effective as native pancreatic beta-cells in terms of restoring normoglycemia. The cells’ effects on the restoration of normoglycemia were similar between the nonadherent induction group and the adherent induction group.Direct check of dewaxed testis sections (Figure 8A,E,I, gray-scale image) with red fluoroscope showed that the CM-DiI-labeled cells mainly distributed in the interstitial tissue between seminiferous tubules in the graft (Figure 8B,F,J). The immunofluorescent examination of the dewaxed sections indicated that the CM-DiI-labeled red cells in interstitial tissue were positive to human insulin in the nonadherent induction group (green, Figure 8C) and the adherent induction group (green, Figure 8G) instead of those in the non-induction control group (Figure 8K), displaying that the insulin was produced by the transplanted islet-like cell clusters instead of by non-induced hfBMSCs.

## Discussion

### Nonadherent induction strategies and principle

BMSCs are a type of cells with strong adhesion tendency. Inducing such cells into cell clusters in lysine-coated plastic dishes is difficult. In this study, hfBMSCs in noncoated plastic dishes (under a nonadherent state) were more efficiently induced into functional pancreatic islet-like cell clusters than those in lysine-coated dishes (under an adherent state) using a three-stage induction procedure. The reason for this difference might be that nonadherent induction in noncoated dishes not only facilitated the contact of hfBMSCs with inducers but also provided a good opportunity for the chemotactic movement, aggregation and clustering of hfBMSCs. Cells in cell clusters, affected by surrounding cells via paracrine, easily coordinated each other and differentiated. Chang and colleagues showed that human BMSCs centrifuged into cell pellets more easily differentiated towards IPCs than those under a monolayer [8]. Studies have shown that cell–cell contact between pancreatic beta-cells is important for maintaining their survival and increasing insulin secretion [24]. In the present study, there were IPCs only in the islet-like cell clusters, whereas single cells in adherent growth did not differentiate towards target cells under the same induction according to their shape and immunofluorescence assay (Figure 4), proving that the environment within the cell clusters was able to promote cell differentiation.

Nestin, a marker of neural stem cells, is also expressed by cells located in the epithelium of the pancreatic primordium. Nestin-positive cells generate the cells of the endocrine and exocrine lineages [25,26], and nestin-positive stem cells from mouse embryonic stem cells can be induced into insulin-positive and c-peptide-positive cells [27]. The newest research indicated that nestin plays pivotal roles as an intermediate regulator governing both stemness and differentiation of stem cells in the process of their differentiation into IPCs [28]. In this study, we efficiently induced hfBMSCs first into nestin-positive multipotential cell clusters, then into pancreatic islet-like cell clusters and finally into functional islet-like cell clusters (Figures 4 and 6) in noncoated plastic dishes using the three-stage induction procedure. However, the characteristics of nestin-positive cells are still debatable.

### Differentiation effects of hfBMSCs after nonadherent induction

In this study, 65.08 ± 2.98% of the hfBMSCs under nonadherent induction differentiated into pancreatic islet-like cell clusters, which is significantly higher than their clustering rate (42.37 ± 3.70%) under adherent induction (*P* < 0.01, *n* = 10). The differentiation rate of hfBMSCs towards IPCs is 29.80 ± 3.95% in the nonadherent induction group, which is significantly higher than the differentiation rate (18.40 ± 2.08%) in the adherent induction group (*P* < 0.01, *n* = 10), as well as higher than the differentiation rate (10 to 20%) of mouse BMSCs in adherence towards IPCs reported by Tang and colleagues [4] and the differentiation rate (19.8%) of rat BMSCs in adherence towards IPCs reported by Wu and colleagues [6]. These results indicate that nonadherent induction in noncoated plastic dishes can greatly promote BMSCs to form pancreatic islet-like cell clusters, thereby improving the differentiation rate of BMSCs towards IPCs.

In comparison with the fetal pancreatic islets, the islet-like cell clusters from hfBMSCs expressed more Ngn3 and Pax4, the markers of early stage beta-cell development, and secreted relatively lower insulin, glucagon, SST and PP (all *P* < 0.01, *n* = 10), indicating that their differentiation was not full *in vitro*.

### Effects of islet-like cell clusters on mouse diabetic treatment

Although some attempts have been made to treat diabetes in an animal model with induced BMSCs in adherence, the results are not ideal. Wu and colleagues reported that the glucose levels of STZ-induced diabetic rats began decreasing at day 6 after the allograft with 5 × 10^6^ induced rat BMSCs in adherence, and remained below 15 mmol/l from day 12 to day 16 post transplantation but elevated again after day 20 [6]. The research by Hisanaga and colleagues showed that the plasma glucose level of STZ-induced diabetic mice markedly fell after the allograft with 5 × 10^7^ induced mouse BMSCs in adherence and the effect continued for at least 4 weeks [7]. Xie and colleagues ameliorated the diabetic conditions of STZ-treated mice by the transplantation of IPCs derived from BMSCs [9]. In this study, the hyperglycemia of diabetic nude mice was normalized by transplanting 600 pancreatic islet-like cell clusters (about 7.2 × 10^5^ cells) from hfBMSCs. The blood glucose levels of three mice rose again after removal of the grafts while seven mice without removal of the graft maintained normal blood glucose levels for at least 80 days (Figure 7A), indicating that the transplanted cells were responsible for the cure of hyperglycemia. This observation was further confirmed by colabeling of CM-DiI and immunofluorescent antibodies against human insulin in the grafts, which demonstrated that the transplanted cells produced functional human insulin *in vivo* (Figure 8).

In addition, in this study the islet-like cell clusters were transplanted in the testes of model animals because not only there is a strong blood circulation in the testis but also operation of cell transplantation and withdrawal of the graft aimed at the testis is easier than that aimed at other organs, such as a renal subcapsular graft. The transplanted islet-like cell clusters normalized the blood glucose levels of diabetic model mice 12 days after transplantation (Figure 7A), implying that they might go through a process of adaptation, proliferation, maturation and gradual functioning *in vivo*.

### BMSCs from human first-trimester abortus

We are not advocating carrying out abortions for the single purpose of obtaining human fetal BMSCs, but a human abortus is a source of obtaining a variety of fetal stem cells, especially in some countries with family planning policy or birth-control policy. Certainly, such an approach may be controversial in some other countries. The properties of hfBMSCs range between those of embryonic stem cells and adult stem cells, but hfBMSCs form no tumors in mice [29] whereas embryonic stem cells induced towards IPCs still form tumors in mice [30,31]. Thus hfBMSCs are an ideal type of seed cell for research on human tissue engineering and regeneration medicine.

## Conclusions

This study demonstrates that nonadherent induction can greatly promote BMSCs to form pancreatic islet-like cell clusters, thereby improving the differentiation efficiency of BMSCs towards IPCs.

## Abbreviations

BMSC: bone marrow mesenchymal stem cell; BSA: bovine serum albumin; DMEM: Dulbecco’s modified Eagle’s medium; EGF: epidermal growth factor; hfBMSC: bone marrow mesenchymal stem cell of human first-trimester abortus; IPC: insulin-producing cell; PBS: phosphate-buffered saline; STZ: streptozocin.

## Competing interests

The authors declare that they have no competing interests.

## Authors’ contributions

YZ participated in the design of the study, carried out the whole experiment, performed the statistical analysis of data, drafted the manuscript and approved the final manuscript. ZD participated in the design, coordination and the revision of the manuscript, and approved the final manuscript.
